# Does voluntary hypoventilation during exercise impact EMG activity?

**DOI:** 10.1186/s40064-016-1845-x

**Published:** 2016-02-24

**Authors:** Daisuke Kume, Shogo Akahoshi, Takashi Yamagata, Toshihiro Wakimoto, Noriki Nagao

**Affiliations:** Department of Integrated Arts and Science, National Institute of Technology, Okinawa College, 905, Henoko, Nago, Okinawa 905-2192 Japan; Department of Health and Sports Science, Kawasaki University of Medical Welfare, 288, Matsushima, Kurashiki, Okayama 701-0193 Japan; Department of Clothing, Japan Women’s University, 2-8-1, Mejirodai, Bunkyoku, Tokyo 112-8681 Japan; Department of Nursing, Hyogo University, 2301, Hiraokachoshinzaike, Kakogawa, Hyogo 675-0195 Japan

**Keywords:** Hypoventilation, Exercise, Hypoxia, Muscle oxygenation, Electromyography

## Abstract

It has been reported that exercise under hypoxic conditions induces reduced muscle oxygenation, which could be related to enhanced activity on electromyography (EMG). Although it has been demonstrated that exercise under conditions of voluntary hypoventilation (VH) evokes muscle deoxygenation, it is unclear whether VH during exercise impacts EMG. Seven men performed bicycle exercise for 5 min at 65 % of peak oxygen uptake with normal breathing (NB) and VH. Muscle oxygenation; concentration changes in oxyhemoglobin (Oxy-Hb), deoxyhemoglobin (Deoxy-Hb) and total hemoglobin (Total-Hb); and surface EMG in the vastus lateralis muscle were simultaneously measured. In the VH condition, Oxy-Hb was significantly lower and Deoxy-Hb was significantly higher compared to those in the NB condition *(P* < 0.05 for both), whereas there was no significant difference in Total-Hb between the two conditions. We observed significantly higher values *(P* < 0.05) on integrated EMG during exercise under VH conditions compared to those under NB conditions. This study suggests that VH during exercise augments EMG activity.

## Background

Previous studies by Woorons et al. (2007, 2010, 2011) demonstrated that voluntary hypoventilation (VH) during bicycle exercise elicits a marked drop in arterial oxygen saturation (SaO_2_). A recent study Woorons et al. (2014) also revealed that VH during swimming exercise can lead to severe arterial desaturation. These results suggest that exercise training under conditions of VH can result in hypoxia. Exercise training at a simulated altitude using a hypoxic chamber is an accepted method for improving endurance capacity (Dufour et al. 2006; Hendriksen and Meeuwsen 2003), and the use of “artificially induced hypoxic conditions” by an athlete is not prohibited by the World Anti-Doping Agency (Levine 2006). However, in practice, only a few institutions provide the hypoxic system for training and the devices which create a hypoxic environment are very expensive. Thus, VH hypoxic training might be a practical alternative method for many athletes.

It has been well-documented that exercise under hypoxic conditions evokes reduced muscle oxygenation as measured by near-infrared spectroscopy (NIRS) (Kawahara et al. 2008; Subudhi et al. 2007, 2008). It has also been reported that hypoxia augments activity on electromyography (EMG) during exercise (Amann et al. 2006, 2007; Taylor et al. 1997; Katayama et al. 2006, 2010). Katayama et al. (2010) conducted a study using two types of isometric quadriceps exercise, which were either sustained or intermittent exercise, under normoxic and hypoxic conditions. Consequently, both muscle deoxygenation and EMG responses were exaggerated during intermittent isometric exercise under hypoxic compared with normoxic conditions, whereas there were no differences between conditions for either muscle oxygenation or EMG during sustained exercise. Therefore, it is thought that hypoxia-induced muscle deoxygenation may affect the rate of accumulation of muscle metabolites, such as lactate and inorganic phosphate (Pi) (Hogan et al. 1999; Richardson et al. 1998), which leads to increased motor unit recruitment (Amann and Calbet 2008). On the other hand, Woorons et al. (2010) reported that exercise under VH conditions elicits lower muscle oxygenation compared to that during exercise with normal breathing (NB). We also observed decreased muscle oxygenation levels during exercise under VH conditions in our preliminary experiments. These findings led to the hypothesis that exercise under conditions of VH, which provokes muscle deoxygenation, could result in enhanced EMG. However, the impact of VH during exercise on EMG activity is unclear.

The purpose of this study was to elucidate the impact of VH during exercise on EMG activity. To accomplish this, we determined muscle oxygenation and surface EMG detected from the vastus lateralis muscle during bicycle exercise under conditions of VH and NB.

## Methods

### Subjects

Seven healthy, active men participated in this study (mean ± SD: age, 23.4 ± 3.2 years; height, 174.4 ± 2.1 cm; body weight, 66.8 ± 3.5 kg). All subjects were engaged in physical activity 3–4 times a week. Informed consent was obtained from each participant after they had received verbal and written explanation of the experimental procedures and possible risks. This study was approved by the Ethics Committee of Kawasaki University of Medical Welfare prior to the initiation of experiments.

### Experimental overview

All subjects performed a maximal exercise test using an electrically braked cycle ergometer (Aerobike 75XLiii; Combi, Tokyo, Japan) to determine their peak oxygen uptake (${\dot{\text{V}}}$O_2peak_). Participants came once or twice to the laboratory to familiarize themselves with the equipment as well as with the VH technique before starting the next tests. VH practices were performed with exercising at an intensity corresponding to 65 % of ${\dot{\text{V}}}$O_2peak_. About 1 week after the maximal exercise test, individuals performed two submaximal exercise tests under the VH and NB conditions. The tests were separated by 60 min to allow a rest period. The order of exercise conditions was randomly assigned and counterbalanced. Both tests were performed at least 2 h after a light meal. Moreover, all subjects were asked to refrain from caffeine-containing beverages and to avoid strenuous exercise for 12 h before each experiment.

### Maximal exercise test

The seat height of the ergometer was adjusted so that there was a slight bend in the knee joint when the foot pedal was at its lowest point. After a 5-min rest on the cycle ergometer, the exercise test began at an initial power output of 90 W for 4 min, and the load was then increased by 30 W every 2 min until exhaustion. The pedaling rate was maintained at 60 rpm with the aid of a metronome. Subjects were verbally encouraged to perform at maximum effort. The test was terminated when the subject failed to maintain the prescribed pedaling rate. The highest ${\dot{\text{V}}}$O_2_ value obtained throughout the exercise protocol was used as the ${\dot{\text{V}}}$O_2peak_.

### Submaximal exercise test

The seat height of the ergometer was adjusted to the same position previously used by each subject. After a 5-min rest on the cycle ergometer, subjects performed a 3-min warm-up exercise at an exercise intensity corresponding to 45 % of ${\dot{\text{V}}}$O_2peak_ with NB, followed by a 5-min exercise at 65 % ${\dot{\text{V}}}$O_2peak_ under conditions of VH or NB. We employed the modified VH technique reported by Woorons et al. (2007). Namely, when the exercise was performed under VH conditions, the 5-min exercise was divided into five periods of 1 min each that included 10 s of NB followed by 50 s of VH. During the VH periods, subjects expired continuously and progressively for 4 s down to near residual volume and then inspired quickly, and the sequence was repeated. Muscle oxygenation and data from surface EMG were recorded during the exercise continuously. Also, such respiratory gas parameters as minute ventilation (${\dot{\text{V}}}$E), ${\dot{\text{V}}}$O_2_, carbon dioxide output (${\dot{\text{V}}}$CO_2_), respiratory exchange ratio (RER), heart rate (HR) and SaO_2_ were continuously recorded throughout the tests. Moreover, at the rest period and immediately after cessation of exercise, blood lactate (BLa) levels were determined.

### Measurement

Respiratory gases were measured using an automatic gas analyzer (VO2000; MedGraphics, Minneapolis, USA), and the sampling time was set at 30 s. HR was monitored at 5 s intervals using a wrist HR monitor (RS800CX; Polar Electro Oy, Kempele, Finland). SaO_2_ was measured every second using a pulse oximeter (WEC-7201; Nihon Kohden, Tokyo, Japan). BLa was determined from finger capillary blood samples using an automatic lactate analyzer (Lactate Pro LT-1710; Arkray Inc., Kyoto, Japan).

### NIRS

Tissue oxygenation in the right vastus lateralis muscle, which is the main working muscle in the leg during cycling exercise (Ericson et al. 1985), was measured using a continuous-wave NIRS system (PSA-500; Biomedical Science, Kanazawa, Japan). This apparatus was used in previous our study (Kume et al. 2013). The device uses two-wavelength light emitting diodes (750 and 830 nm) as the light source, and measures concentration changes in oxyhemoglobin (Oxy-Hb) and deoxyhemoglobin (Deoxy-Hb), based on the modified Beer-Lambert law. The sum of these variables reflects concentration changes in total hemoglobin (Total-Hb). Measurement values from our device are expressed in grams per liter per centimeter, which was converted from moles per liter per centimeter as a unit of molar absorbance coefficient. The NIRS signals were sampled at 1 Hz. The distance between the light source and the detector in the NIRS probe was 30 mm. The depth of the measured area was approximately half of the distance between the light source and the detector, i.e., about 1.5 cm (McCully and Hamaoka 2000). Skinfold thickness at the probe site was determined using an ultrasonographic system (SDU-500; Shimadzu Corporation, Kyoto, Japan) to confirm the measurement area for the NIRS: with skin thicknesses of 5.1 ± 0.9 mm, the light penetration depth was adequate. The probe was firmly positioned at a point one-third of the way from the patella to the greater trochanter (Kime et al. 2009) using double-sided tape and covered with an elastic bandage to minimize movement. NIRS variables during the 5-min exercise were expressed as delta scores by subtracting the mean values recorded during the first minute of the warm-up exercise with NB (ΔTotal-Hb, ΔOxy-Hb and ΔDeoxy-Hb) (Kume et al. 2013).

### Surface EMG

Surface EMG was monitored on the same muscle used to measure tissue oxygenation. Standard surface electrodes (Blue Sensor; Ambu, Copenhagen, Denmark) were used as the recording electrodes and were placed over the distal side of the NIRS probe, parallel to the longitudinal axis of the muscle. The interelectrode distance was 20 mm. The ground electrode was placed on the head of the fibula. The skin surface was shaved and cleaned with alcohol and abraded with sandpaper. To avoid movement-induced artifact, the wires connected to the electrodes were secured with tape. EMG recording was conducted using a surface EMG instrument (Personal-EMG; Oisaka Electronic Device, Hiroshima, Japan). The EMG signals were sampled at a frequency of 1000 Hz, and the signals from two pairs of electrodes were amplified 1000 times. Band-pass filtering was set at 20–500 Hz. The EMG was full-wave rectified and integrated (iEMG) automatically. EMG measurement settings and treatments were performed using commercially available software (Personal-EMG software, Oisaka Electronic Device, Hiroshima, Japan). The percent changes in iEMG from first minute the 5-min exercise were calculated (ΔiEMG) (Amann et al. 2006, 2007).

Measurement variables that were continuously recorded during the 5-min exercise were averaged every minute.

### Statistical analysis

Data are expressed as mean ± SD. Differences in the changes of the measurement variables between the conditions were evaluated by a two-way analysis of variance with repeated measures followed by the Bonferroni post hoc test. SPSS statistical software (version 19.0; SPSS, Tokyo, Japan) was used for analysis. The level of statistical significance was set at *P* < 0.05.

## Results

### Maximal exercise test

Cardiorespiratory variables and workload at exhaustion during maximal exercise testing are as follows: ${\dot{\text{V}}}$O_2_, 3.49 ± 0.19 L/min, 52.2 ± 2.4 mL/kg/min, ${\dot{\text{V}}}$E, 135.0 ± 13.1 L/min, ${\dot{\text{V}}}$CO_2_, 3.84 ± 0.22 L/min, RER, 1.10 ± 0.04, HR, 191 ± 7 beats/min, SaO_2_, 94.0 ± 1.9 %, and workload, 282.9 ± 16.0 W.

### Submaximal exercise test

#### Cardiorespiratory variables

The results are presented in Table 1. ${\dot{\text{V}}}$E during exercise in the VH condition was significantly lower (*P* < 0.05) than that in the NB condition. ${\dot{\text{V}}}$O_2_ in the VH condition tended to be lower than that in the NB condition, although the difference was not significant. ${\dot{\text{V}}}$CO_2_ was significantly lower (*P* < 0.05) at 1 and 2 min in the VH condition than that in the NB condition. There were no significant differences between conditions for RER and HR. SaO_2_ in the VH condition was significantly lower (*P* < 0.05) than that in the NB condition.

**Table 1:** Cardiorespiratory variables during the 5-min exercise

Variables	Conditions	1 min	2 min	3 min	4 min	5 min
${\dot{\text{V}}}$E (L/min)	NB	50.34 ± 3.60	61.60 ± 4.63	69.52 ± 5.30	69.52 ± 5.30	68.46 ± 3.97
	VH	36.14 ± 4.78*	45.27 ± 2.03*	50.58 ± 4.87*	52.52 ± 4.77*	53.60 ± 5.11*
${\dot{\text{V}}}$O_2_ (L/min)	NB	1.93 ± 0.12	2.22 ± 0.11	2.38 ± 0.07	2.41 ± 0.06	2.46 ± 0.05
	VH	1.66 ± 0.14	2.03 ± 0.14	2.20 ± 0.14	2.23 ± 0.23	2.34 ± 0.15
${\dot{\text{V}}}$CO_2_ (L/min)	NB	1.55 ± 0.10	1.88 ± 0.13	2.05 ± 0.14	2.12 ± 0.15	2.08 ± 0.10
	VH	1.24 ± 0.11*	1.69 ± 0.09*	1.83 ± 0.20	1.90 ± 0.20	2.00 ± 0.14
RER	NB	0.81 ± 0.04	0.85 ± 0.04	0.86 ± 0.05	0.88 ± 0.07	0.85 ± 0.04
	VH	0.75 ± 0.02	0.80 ± 0.03	0.83 ± 0.04	0.85 ± 0.03	0.85 ± 0.02
HR (beats/min)	NB	127 ± 7	136 ± 6	142 ± 8	145 ± 7	147 ± 9
	VH	128 ± 6	136 ± 7	142 ± 7	147 ± 7	150 ± 7
SaO_2_ (%)	NB	98.4 ± 0.7	98.0 ± 0.9	97.5 ± 0.7	97.5 ± 0.5	97.4 ± 0.7
	VH	97.2 ± 1.5*	93.2 ± 3.4*	92.3 ± 2.5*	91.2 ± 2.1*	89.6 ± 3.2*

Values are mean ± SD. * *P* < 0.05 vs. NB condition

#### NIRS variables

**Fig. 1:**
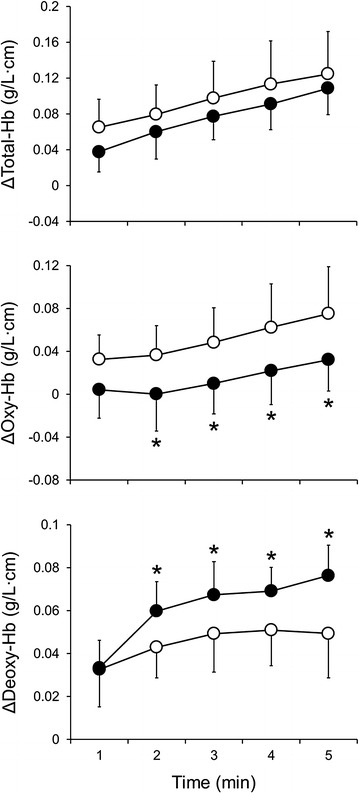
Changes in NIRS variables under NB (*open circles*) and VH (*filled circles*) conditions. Values are mean ± SD. * *P* < 0.05 vs. NB condition

The results are presented in Fig. 1. No significant difference in ΔTotal-Hb was observed between the conditions. At 2–5 min in the VH condition, ΔOxy-Hb was significantly lower and ΔDeoxy-Hb was significantly higher (*P* < 0.05, respectively) than those in the NB condition.

#### EMG activity

**Fig. 2:**
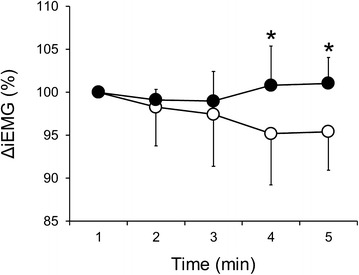
Changes in EMG activity under NB (*open circles*) and VH (*filled circles*) conditions. Values are mean ± SD. * *P* < 0.05 vs. NB condition

The results are presented in Fig. 2. The values for ΔiEMG were significantly higher (*P* < 0.05) at 4–5 min in the VH condition than those in the NB condition.

#### BLa

**Fig. 3:**
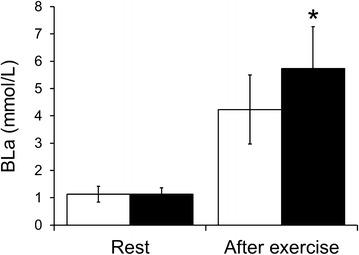
BLa levels at rest and immediately after cessation of exercise under NB (*open bars*) and VH (*filled bars*) conditions. Values are mean ± SD. * *P* < 0.05 vs. NB condition

The results are presented in Fig. 3. There was no significant difference in the resting values between the conditions. After exercise, values were significantly higher (*P* < 0.05) in the VH condition than in the NB condition.

## Discussion

The primary finding of this study was that augmented EMG activity was observed during VH exercise, which was accompanied by muscle deoxygenation. These finding supports our hypothesis that VH during exercise enhances EMG responses.

In the present study, we confirmed the obvious arterial desaturation and muscle deoxygenation during exercise under VH conditions. These results are consistent with those from a previous study (Woorons et al. 2010) and our preliminary observations. Woorons et al. (2007, 2010, 2011) have indicated that exercise under VH conditions is different from exercise under hypoxic conditions because VH also induces hypercapnia. The hypercapnic response leads to respiratory acidosis and consequently a right shift of the oxygen dissociation curve. Thus, VH-induced hypercapnia more facilitates arterial desaturation and muscle deoxygenation than does hypoxia (Woorons et al. 2007). We presume that similar hypercapnic effects occurred in the VH condition in this study, although arterial and end-tidal carbon dioxide pressure measurements were not taken.

It has been reported that hypoxia-induced muscle deoxygenation could be related to enhanced EMG (Katayama et al. 2010). From that report, we hypothesized that VH exercise, which produces muscle deoxygenation, would impact EMG. In this study, the values for ΔiEMG in the VH condition were significantly higher than those in the NB condition. The findings in this study support our hypothesis and suggest that VH during exercise enhances EMG activity. Regarding the possible mechanisms for this relationship, it has been shown that augmented muscle deoxygenation accelerates the accumulation of muscle metabolites, such as lactate and Pi (Hogan et al. 1999; Richardson et al. 1998). In fact, we observed a pronounced increase in BLa by VH during exercise, as shown in Fig. 3; this increased BLa indicates a greater accumulation of muscle metabolites. The accumulation of muscle metabolites causes failure of excitation–contraction coupling within the muscle fiber, resulting in loss of tension development in each muscle fiber (Amann and Calbet 2008; Erdogan et al. 2002). Thus, in the VH condition, to compensate for weakened tension in the muscle, the firing rate and/or recruitment of muscle fibers could have increased. Alternatively, on the basis of the size principle, the additional motor units recruited would have been larger in size and had a higher threshold, like type II fibers. Increased type II fiber recruitment is associated with higher spike amplitudes (Moritani et al. 1986; Taylor et al. 1997). Consequently, EMG activity should have been enhanced during exercise with VH. Another possible reason for enhanced EMG in the VH condition, as mentioned above, is that exercise under VH conditions induces hypercapnia, which is the opposite to exercising under hypoxic conditions (Woorons et al. 2007, 2010, 2011). It has been reported that hypercapnia depresses limb muscle contractility (Mador et al. 1997; Vianna et al. 1990) and that hypercapnia has the potential to produce a greater recruitment of type II fibers (Hilbert et al. 2012). Therefore, we suspect that hypercapnic effects partially contributed to the increased EMG activity.

The present study has at least two limitations: (1) the sample studied was small and (2) surface electrodes on the vastus lateralis could pick up activity from neighboring muscles. These issues must be considered when interpreting the results; however, the limitations do not alter the essential findings of this study.

To conclude, our current results suggest that VH during exercise enhances EMG.

## Abbreviations

BLa: blood lactate
Deoxy-Hb: deoxyhemoglobin
EMG: electromyography
HR: heart rate
iEMG: integrated electromyography
NB: normal breathing
NIRS: near-infrared spectroscopy
Oxy-Hb: oxyhemoglobin
RER: respiratory exchange ratio
SaO_2_: arterial oxygen saturation
Total-Hb: total hemoglobin
${\dot{\text{V}}}$CO_2_: carbon dioxide output
$${\dot{\text{V}}}$$E: minute ventilation
VH: voluntary hypoventilation
$${\dot{\text{V}}}$$O_2_: oxygen uptake
